# The Challenges to Advancing Induced Pluripotent Stem Cell-Dependent Cell Replacement Therapy

**DOI:** 10.18103/mra.v11i11.4784

**Published:** 2023-11-29

**Authors:** Alan B. Moy, Anant Kamath, Sara Ternes, Jay Kamath

**Affiliations:** 1 Cellular Engineering Technologies, Inc. Coralville, IA, 52241; 2 John Paul II Medical Research Institute, Coralville, IA 52241

## Abstract

Induced pluripotent stem cells (iPSC) represent a potentially exciting regenerative-medicine cell therapy for several chronic conditions such as macular degeneration, soft tissue and orthopedic conditions, cardiopulmonary disease, cancer, neurodegenerative disorders and metabolic disorders. The field of iPSC therapeutics currently exists at an early stage of development. There are several important stakeholders that include academia, industry, regulatory agencies, financial institutions and patients who are committed to advance the field. Yet, unlike more established therapeutic modalities like small and large molecules, iPSC therapies pose significant unique challenges with respect to safety, potency, genetic stability, immunogenicity, tumorgenicity, cell reproducibility, scalability and engraftment. The aim of this review article is to highlight the unique technical challenges that need to be addressed before iPSC technology can be fully realized as a cell replacement therapy. Additionally, this manuscript offers some potential solutions and identifies areas of focus that should be considered in order for the iPSC field to achieve its promise. The scope of this article covers the following areas: (1) the impact of different iPSC reprogramming methods on immunogenicity and tumorigenicity; (2) the effect of genetic instability on cell reproducibility and differentiation; (3) the role of growth factors and post-translational modification on differentiation and cell scalability; (4) the potential use of gene editing in improving iPSC differentiation; (5) the advantages and disadvantages between autologous and allogeneic cell therapy; (6) the regulatory considerations in developing a viable and reproducible cell product; and (7) the impact of local tissue inflammation on cell engraftment and cell viability.

## Introduction

Chronic disease resulting from degenerative organ dysfunction accounts for the vast majority of the global healthcare costs ^1^. While organ transplantation is a definitive treatment for several end-staged organ disorders, there is an insufficient supply of available organ donors ^2,3^. Furthermore, the high cost of organ transplantation poses substantial financial strain on national healthcare costs. Stem cell therapy represents a potential alternative solution to the limited availability and high costs of organ transplantation. Regenerative medicine utilizing stem cells offers more likelihood of success in reversing chronic diseases when compared with using small and large molecules. Pluripotent stem cells represent a viable alternative and cost-effective regenerative medicine solution for several chronic conditions such as macular degeneration, soft tissue and orthopedic conditions, cardiopulmonary disease, cancer, neurodegenerative disorders and metabolic disorders. Although human embryonic stem cells (ESCs) represented the first described pluripotent stem cells ^4^, these cells pose specific shortcomings. Notwithstanding the ethical controversy, ESC exhibits a neoplastic propensity and displays genetic instability ^5,6^. Additionally, they pose a risk of host vs. graft rejection from the human leukocyte antigen (HLA) mismatch between donor and recipient. Next, there is an inherent lack of available human embryos from *in vitro* fertilization clinics that are donated for medical research ^7^. Finally, in many instances, the original master cell banks were not created under what is deemed to be current Good Manufacturing Practices (cGMP) ^8^.

Induced pluripotent stem cells (iPSC) pose an alternative and ample source of pluripotent stem cells that can be readily manufactured under cGMP conditions. Yet, unlike small and large molecules, cell therapies pose significant and unique manufacturing and quality-controlled challenges. Small and large molecules offer defined pharmacological characteristics that can be more readily manufactured into reproducible products. However, iPSC, like other stem cells, are much more complex when considering therapeutic application. Induced pluripotent stem cell therapies pose significant unique challenges with respect to safety, potency, genetic stability, immunogenicity, tumorgenicity, cell reproducibility and scalability.

The aim of this review article is to highlight the unique technical challenges that need to be addressed before iPSC technology can be fully realized as a cell replacement therapy. Additionally, this manuscript offers potential solutions and identifies areas of focus that should be considered in order for the iPSC field to achieve its promise. The scope of this article covers the following areas: (1) the impact of different iPSC reprogramming methods on immunogenicity and tumorigenicity; (2) the effect of genetic instability on cell reproducibility and differentiation; (3) the role of growth factors and post-translational modification on cell scalability; (4) the potential use of gene editing in improving iPSC differentiation; (5) the advantages and disadvantages between autologous and allogeneic cell therapy; (6) the regulatory considerations in developing and transporting a reproducible cell product to the point of patient administration; and (7) the impact of local tissue inflammation on cell engraftment and cell viability. This report will focus primarily on the potential use of iPSC technology as a cell replacement therapy for solid organ disease, rather than the application of iPSC in adoptive cell therapy (ACT) for treating cancer. There are several good review articles on iPSC applications in the field of ACT ^9–12^.

### Induced Pluripotent Stem Cell Reprogramming Methods

Induced pluripotent stem cells (iPSCs) represent a noncontroversial source of pluripotent stem cells that can satisfy the requirements of providing an unlimited supply of cGMP production of master cell line stocks. Takahashi et al. were the first to report the dedifferentiation of somatic fibroblasts into pluripotent stem cells by retroviral gene delivery of Oct3/4, Sox2, Klf4 and c-Myc ^13,14^. Yu et al. also reported creating cultured iPSC from fetal and neonatal fibroblasts by retroviral delivery of Oct4, Sox2, Nanog and Lin28 ^15^. Both groups demonstrated that pluripotent stem cells had similar characteristics to those reported in human ESCs. Nakagawa et al. further observed that deletion of c-Myc from the reprogramming scheme still created pluripotent colonies but eliminated teratoma formation in the short-term ^16^. Yet, the authors reported a significantly lower reprogramming efficiency under this condition even when retroviral gene delivery was deployed. Nakagawa et al. published a follow-up report demonstrating that replacement of c-Myc with l-Myc eliminated, or at the very least reduced, the neoplastic effects associated with c-Myc ^17^. While l-Myc does not promote teratoma formation at the same frequency as c-Myc in short-term experiments involving murine models, l-Myc has been associated with several clinical malignancies ^18–20^. Also, heterologous expression of c-Myc as described in this report led to a much lower fraction of fully reprogrammed colonies than those created from heterologous l-Myc expression ^17^. Taken together, the data indicate that the oncogenes, c-Myc and Lin28, are the chief determinants of the neoplastic risk associated with current iPSC reprogramming methods. In addition to the oncogenes that are used in cellular reprogramming, retroviral gene transfer methods also pose an additional risk of genomic integration that could silence important tumor suppressor genes. This could again increase the neoplastic risk, as well as mediate a risk of viral-mediated immunogenicity.

To improve the safety risks associated with viral-mediated reprogramming approaches, subsequent non-viral and/or non-integrating approaches were developed to produce clinical-grade iPSC lines. These included nonviral reprogramming methods such as piggyback ^21^, DNA minicircles ^22^ and microRNA ^23^. However, these methods proved to be extremely inefficient for reprogramming. More efficient non-integrating approaches emerged and the most popular methods include the use of Sendai vectors ^24–27^, mRNA reprogramming ^28–30^, self-replicating RNA ^31^ and episomal vectors ^32,33^. Each of these methods have advantages and disadvantages. Self-replicating or replicon RNA relies on the Venezuelan equine encephalitis virus positive sense, single-stranded RNA backbone and its ability to mimic cellular mRNA without having a DNA intermediate. It is known that to get an appreciable level of reprogramming with self-replicating RNA, transfection with co-agents that suppress immune response are necessary ^31^. Moreover, PCR studies have shown they retain the expression of viral RNA components for at least four passages downstream. This makes iPSC colony selection, especially for therapeutics, a lengthy process. mRNA-based reprogramming ^28–30^ requires repeated daily transfections for up to 17 days and is laborious and expensive. Also, this method must contend with interferon production in transfected cells, which impacts reprogramming efficiency and which could present downstream immunological concerns. Moreover, somatic suspension cells are resistant to repeated transfection processes. While Sendai virus reprogramming is a popular and robust iPSC reprogramming method, a far greater number of cell divisions are required to dilute the cell line free of contaminating viral proteins and the vector ^24^. Ultimately, iPSC colonies would have to be carefully screened for viral proteins or the viral vector before being selected for a cell therapy. Thus, whatever reprogramming efficiency Sendai viral vectors provide at the beginning is offset by the downstream additional quality control measures that must be implemented to confirm safety.

Episomal reprogramming is an ideal method for creating clinical-grade, safer, nonviral and nonintegrating iPSC. According to the Global Alliance for iPSC Therapies, episomal reprogramming is the most common approach used to produce clinical-grade iPSC because of the rapidity in which the transgene is cleared from targeted cells ^34^. Episomal vectors are only active, on average for 17–21 days, before reaching an undetectable level due to dilution and instability caused by cell division. However, episomal reprogramming efficiency is quite low compared with other nonintegrating methods. To compensate for the lower reprogramming efficiency, others have utilized c-Myc or a combination of l-Myc and Lin28 ^32,35,36^. Yu et al. previously reported no colony formation using an episomal reprogramming strategy that delivered Oct4, Sox2, c-Myc, Nanog, Lin28 and Klf4 ^33^. However, the authors reported colony formation at an efficiency of approximately 0.0006% only after the addition of an SV40 large T-antigen gene. In contrast, Okita et al. previously reported a similar quantitative reprogramming efficiency from episomal-derived IPSC colonies with p53 suppression combined with l-Myc and Lin28 heterologous expression ^32^.

We first reported a method using episomal vectors without using oncogenes (defined as free of *Lin28*, *Myc*, Nanog and SV40) in iPSC reprogramming in adherent cultured human foreskin fibroblasts (HFF) by using a mixture of reprogramming small molecules ^37^. The use of these reprogramming molecules produced fully reprogrammed iPSC colonies even in the absence of *c-Myc* or a combination of *l-Myc/Lin28*. Virtually, 100 percent of the colonies were fully reprogrammed based on SSEA4 expression, which exceeds the 70 percent threshold recommended by the Global Alliance in iPSC Therapy ^34^. Moreover, our reprogramming method lowers the neoplastic risk and eliminates the viral immunogenicity risk. We subsequently reported how the same method with minor adjustments produced reproducible iPSC cells from suspension cells ^38^. We documented iPSC from purified cord blood derived CD34+ cells, cord blood derived mononuclear cells and peripheral blood mononuclear cells from patients with cystic fibrosis and alpha 1 anti-trypsin deficiency by first pre-treatment of cells with thrombopoietin ^38^. Induced pluripotent stem cell colonies formed in the absence of oncogenes and virtually 100 percent of the colonies were fully reprogrammed as defined by the expression of SSEA4. Thus, our iPSC reprogramming method is reproducible, relatively quick, universal for both adherent and suspension cells, free of the need to screen colonies for pluripotency, and meets a high criterion for safety. Pluripotent stem cell-based therapy requires purification of the differentiated cell from residual pluripotent stem cells. However, purification processes in bio-manufacturing rarely achieves 100 percent. Even if a final cell differentiation product could be purified from residual pluripotent stem cells at a 99 percent level, there would be 700,000–1,400,000 residual pluripotent stem cells at typical adult systemic dose of 1–2 million cells/kilogram of body weight. Lee previously reported that it only took 10,000 human pluripotent stem cells to form teratomas when injected into murine skeletal muscle and 100,000 cells to form teratomas when injected into mouse hearts ^39^. The neoplastic risk could potentially increase with immune-tolerant allogeneic iPSC if the intrinsic neoplastic risk is not reduced because the resulting cell therapy could evade the recipient’s ability to immunologically-clear undifferentiated pluripotent stem cells.

### Genetic Instability of Induced Pluripotent Stem Cells

Pluripotent stem cells exhibit significant genetic instability ^5,6^. Genetic instability typically results from non-random mutations on chromosomes 1,12,17 and 20 ^5,6^. It is well reported that c-Myc leads to genetic instability in cultured iPSC ^40^. Most of these genetic changes are due to gains rather than losses in genetic material ^5^. Genetic modifications on chromosomes 12 and 17 result in increased clonal proliferation. This imposes selective pressure that promotes the expansion of genetic variants thereby increasing the heterogeneity of iPSC cultures and which leads to decreased iPSC reproducibility. Further, genetic instability of iPSC may impair cell differentiation ^6^. Conversely, cell differentiation may further increase genetic instability because of the prerequisite for additional cell expansion of sufficient iPSC before subjecting cells for differentiation processes. Thus, iPSC with preexisting genetic instability may be vulnerable to further genetic instability upon differentiation. Induced pluripotent stem cell-associated genetic instability includes copy number variants and single nucleotide variants in which the latter introduces point mutations in coding and noncoding gene sequences ^6^. Genetic instability is caused by a shift from oxidative respiration to oxidative glycolysis, which leads to increased reactive oxidative stress. Increased oxidative stress leads to impaired DNA repair, mutations and double stranded DNA breaks. Genetic instability is linked to genomic integrated reprogramming methods; prolonged *in vitro* cell passaging; and oncogene reprogramming factors (particularly c-Myc) ^6,40,41^. While karyotyping is typically used to evaluate cytogenetic mosaicism in pluripotent stem cells, the method is insensitive to detecting genetic instability. Multi-gene microarrays, whole genomic sequencing and qPCR are more accurate in detecting genetic instability than karyotyping ^5,6^.

More importantly, little is known about how large-scale production of iPSC and differentiated protocols can impact genetic instability, cell heterogeneity and reproducibility. Quantitative analytical and computational approaches will be required to better define genetic instability and define cell phenotype. The extent of cell culturing conditions and scale-up production variables that lead to genetic instability of iPSC have not been defined, which ultimately could impact the ability to produce a reproducible cell product. Taken together, the need for quality controls in iPSC and differentiated cell manufacturing to create reproducible, safe and potent cell lines that minimize deleterious cell mutations will require high quality tissue culture conditions, computational models and avoidance of oncogenes that are used in reprogramming schemes.

### The Impact of Growth Factors in Induced Pluripotent Stem Cell Therapeutic Development and Cell Scalability

Growth factors represent a seminal role in iPSC reprogramming and *ex vivo* expansion. Transforming growth factor -beta and fibroblast growth factor are typically used in these processes ^37^. It is important to point out that iPSC transformation into terminally-differentiated cells, which is the ultimate goal in developing a therapeutic cell line, will require multiple differentiation steps, with each step requiring multiple growth factors ^42–47^. Since human cell differentiation under *in utero* conditions is regulated, in part, by native human growth factors, this raises the question of whether manufacturing processes that produce iPSC-differentiated cell therapies should ideally use growth factors that reflect the native chemical structure of these peptides that are operating under *in utero* conditions.

Many current commercial growth factors are manufactured from bacteria. However, bacteria lack a post-translational modification (PTM) system that incorporate glycosylated moieties. To circumvent this dilemma, the bacterial-derived growth factors are truncated to eliminate the glycosylated peptide sequence in order to manufacture that product. Additionally, the PTM pattern produced from cells is species specific. There are differences in the PTM pattern between non-mammalian cells and human cells ^48–50^. Glycosylation affects protein folding, stability, solubility, protein–protein interactions, bioavailability, bio-distribution, pharmacokinetics, immunogenicity and protein activity ^51–58^. There are several reports that document the glycosylation of several growth factors ^59–61^. Moreover, there is little comparative data on iPSC reprogramming and differentiation in the presence of growth factors produced from bacteria, yeast, mammalian cells and human cell lines.

While HEK293 is an established human cell line that manufactures human proteins ^62^, HEK293 poses important shortcomings. Notwithstanding that the cell is ethically controversial because it was derived from an aborted fetus ^63^, HEK 293 cells have an aneuploidy karyotype ^64^. HEK 293 cells were transformed by transfection with adenovirus 5’DNA ^65^. Subsequent analysis has shown that the transformation was formed by inserting 4.5 kilobases from the left arm of the viral genome that was incorporated into chromosome 19 ^66^. There are differences in glycosylation pattern in proteins between Chinese Hamster Ovary (CHO) cells, HEK293 cells and human plasma ^67^. For example, the glycosylation pattern for factor VII when produced from HEK293 cells differs significantly from plasma-derived factor VII ^68^. It not known whether growth factors that display a more native PTM profile, would produce a more potent iPSC-derived cell therapy because the chemistry may more accurately reflect the differentiating activity under *in utero* conditions. In summary, standardized manufacturing processes will not be achieved until a rigorous comparison of cell characterization with growth factors manufactured from different cell sources.

A challenge in developing iPSC-derived cell therapies that may not be fully recognized is the high cost of research and development for producing prototypes during large scale manufacturing. Before a final product is available for preclinical and clinical testing, it is anticipated that a workflow for scaling up a prototypic product must be first optimized. This will initially occur under a less expensive Good Laboratory Practice (GLP), before transferring that process to occur under cGMP conditions, as is required for preclinical and clinical testing. The highest consumable cost in GLP-specific cell product development is the high cost of growth factors from supply chain providers.

Consider the following example. Our group has developed a large-scale workflow of differentiating iPSC into neuroprogenitor cells (NPC), which require Activin A, Noggin, Wnt3a and brain derived neural growth factor (BDNF). We produced these neural growth factors from immortalized human somatic stem cells (PCT/US2023/065911) in order to create peptides that would best approximate the native human PTM system. The following table (Table 1) represents the minimum mass of each neural growth factor required to differentiate 100 million iPSC into approximately 70 million NPC. If commercial supply chains for these growth factors were instead used (which are typically produced from non-human cell lines), then the estimated cost for the mass of growth factors required to produce 70 million NPC would add up to a final cost of $211,655.

Thus, the cost of growth factors to scale-up 700 million and 7 billion NPC would increase by a factor of 10 and 100 respectively. These costs are not sustainable for prototype development. For Parkinson’s disease, the cell therapeutic dose is 4.8 million dopaminergic cells ^69^ where 70 million NPC would provide sufficient dosages for 14 subjects to carry out a Phase 1 clinical trial. In turn, the bioprocessing requirements for Phase 2 and Phase 3 clinical trials would increase from 8 milligrams per growth factor for a Phase 1 clinical trial product to 8 grams per growth factor for a Phase 3 clinical trial product. Additional growth factors will be necessary if the final objective is to further differentiate a NPC into a dopaminergic neuron ^70^ for treating Parkinson’s disease, which would further increase the manufacturing cost. Moreover, the final cost of growth factors produced under cGMP conditions will be even higher than those manufactured under GLP conditions. Thus, there may be a need to not only provide higher quality growth factors that better reflect the native human PTM system, but those research costs have to be substantially reduced to remove financial barriers to develop prototypic iPSC-derived products.

Consistent with the notion that the development costs for cell therapies are challenging, the biology and potency of cell therapies may not be reproducible from small scale to large scale like small and large molecules. Cell behavior is unpredictable during scale-up, which may require cell manufacturers to have the capacity to scale-up and analyze enough cells for a Phase 3 clinical trial even if a Phase 1 clinical trial is the initial objective. The cell phenotype would have to be subjected to rigorous preclinical assays to provide ample evidence that the cell properties are reproducible during the entire scale-up spectrum from a Phase 1 to Phase 3 clinical trials. Industry would have to document that the manufacturing processes from small scale to large scale-up processes produced a consistent and reproducible cell product. Thus, the stakeholders of cell therapies may have to be prepared for significant up-front preclinical research development costs in manufacturing and testing than stakeholders developing small and large molecule therapeutics. Contract manufacturers and contract research service organizations would require more integrative capacity in cell manufacturing and bioprocessing; along with novel and complex cell assay expertise to quantify cell potency; capacity to document quality controls; and the ability to contain costs and provide sufficient services to their biopharmaceutical clients. Taken together, stakeholders that are pursuing cell therapy will be subjected to greater financial, scientific and regulatory risk than those pursuing traditional small and large molecule platforms. Thus, these challenges may restrict cell therapy development to a specialized industry sector because the prerequisite requirements are so great.

### Quality Controls and Regulatory Hurdles in Induced Pluripotent Stem Cell Therapies

Given the complexity associated with cell therapies, iPSC-derived therapies require rigorous quality controls at multiple stages from patient recruitment, tissue procurement, cell isolation, *ex vivo* cell expansion and differentiation, cell phenotype characterization, cell cryopreservation, and finally cold chain management. In the United Sates cellular therapy products are regulated under 21 CFR Part 1271 (Human Cells, Tissues, and Cellular and Tissue-Based Products) ^71^, 21 Part 600 (Biological Products: General) ^72^ and 21 CFR Part 610 (General Biological Products Standards) ^73^. Drug manufacturing requirements are defined in 21 CFR Part 211 (Current Good Manufacturing Practice) ^74^. In Europe cell therapy is regulated by the European Medicines Agency ^75^. A careful medical history of donors is required to confirm that donors are healthy and free of transmissible disease. Procured tissue require testing to confirm that the cell product is free of adventitious disease such as HIV and hepatitis. The Global Alliance in iPSC Therapy has established standard guidelines for developing iPSC therapies ^34^. These guidelines included recommendations for testing genetic fidelity and stability, cell potency and other cellular characteristics. Lastly, *in vitro* bioassays along with computational models are needed to evaluate cell potency to provide an important quality control in monitoring the scale-up process in cell manufacturing.

Once cell products are finally manufactured, additional challenges include implementing cryopreservation methods that provide on-demand access to biological materials, which must mitigate against cryopreservation-induced delayed-onset cell death ^76–79^. Lastly, cold chain management is critical to maintain temperature-sensitive cell therapies during transit to its final destination for patient administration ^80^. Failure in these latter processes can lead to a loss of viable cells that could affect the efficacy of a cell therapy.

### The Inefficiency of Induced Pluripotent Stem Cell Differentiation and Potential Solutions with Gene Editing

Induced pluripotent stem cell differentiation requires multiple steps, and each step requires multiple growth factors. However, the transformation efficiency at each step is frequently inefficient to the extent that only a minority of cells successfully differentiate ^42,43^. Transfection efficiency has improved by the use of gene editing methods by incorporating fluorescent tags or other biomarkers that can enrich differentiated cell species through fluorescence-activated cell sorting (FACS) or by magnetic cell sorting at critical steps.

Gene editing is a rapidly evolving field with multiple methods available to alter cellular DNA. The most widely adopted methods include Clustered Regularly Interspaced Short Palindromic Repeats (CRISPR) and Transcription Activator Like Effector Nucleases (TALENS). CRISPR Cas9 has been shown to be an effective tool for cellular reprogramming applications due to its multiplexing capabilities as well as its ability to target endogenous loci ^81^. The CRISPR Cas 9 gene system makes use of an inactivated Cas9 protein which is recruited to specific stretches of the genome specified by short guide RNA (gRNA) molecules ^82^. The ability of the Cas9 effectors to control the transcription of specific endogenous loci makes it useful to facilitate cellular reprogramming which is predicated on precise activation and silencing of endogenous genes which leads to the proper conversion of cells ^81^. There are significant advantages to using CRISPR for iPSC reprogramming. First, this system has consistently shown high efficiency in making targeted gene edits ^83^. The guide RNA sequences are simple to design and directly target specific genes ^83^. This method is also relatively cost-effective compared to TALENS. ^83^ However, there are currently limitations to CRISPR that must be considered. The current CRISPR Cas9 system limits site selection. In this regard, there are no barriers for gene knockouts, but it may present difficulties when trying to knock in genes at specific locations (e.g. genetic safe harbors) ^84^. Additionally, the extent of off-target effects of CRISPR remains largely unknown. Adopting this technology for large scale reprogramming will require determining whether potential off-target effects will affect the efficiency or downstream efficacy of the reprogramed cells ^84^.

In contrast to CRISPR which is based on site-specific RNA protein interactions, the TALEN gene editing system recognizes a target site based on DNA-protein interactions. Differing associations of the TALE proteins with numerous functional domains such as endonucleases, transcriptional activators and repressors as well as endonucleases allow them to function as both transcriptional modulators as well as gene editing tools ^85^. TALENs have been shown to exhibit high degrees of specificity and low cytotoxicity in numerous cell types. This makes them an attractive complement to more recent CRISPR technologies which currently experience high rates of off-target effects ^86^. However, unlike CRISPR, TALENs are significantly more difficult to design which has limited their widespread adoption in research settings ^85^. Given their ubiquitous use for other genome editing applications, it is anticipated that these gene editing strategies will quickly be adapted to iPSC reprogramming technologies and will improve the differentiation efficiency and decrease the time and cost of producing iPSC-derived cell therapies.

### Autologous vs Allogeneic Induced Pluripotent Stem Cell Considerations

Induced pluripotent stem cell therapies can be either autologous or allogeneic derived. As previously described, the regulatory requirements for allogeneic cell therapy is more demanding than autologous cell therapy. There may be clinical conditions where an autologous cell therapy could have an advantage over allogeneic cell therapies. For example, an autologous natural killer cell (NKC) therapy may have an advantage in treating cancer when cancer cells no longer express major histocompatibility complex (MHC) class 1 antigens ^87^. In contrast, it may be challenging for an allogeneic iPSC-derived NKC to distinguish between a cancer cell that displays low MHC class 1 antigens and non-cancer cells that express a foreign surface antigen. Thus, there may be greater risk for off-target cell injury with an allogeneic-derived NKC therapy.

Autologous iPSC therapies have the advantage for achieving a HLA match between recipient and donor without the need for anti-rejection medication. However, there are important disadvantages. First, the cost for a personalized cell therapy may be more expensive for the healthcare system to pay for such treatment. Second, the target cell for an adult could acquire somatic mutations over the lifespan of a patient that could pose a neoplastic risk. Third, autologous cell replacement therapy for genetic disorders is more expensive and time consuming to develop because putative mutations require corrective gene editing. Lastly, there is greater statistical variability in iPSC phenotypes because of genetic differences between subjects ^88^. Systematic review of hundreds of different iPSC lines showed that 5–46 percent of the cell phenotype differences were due to inter-subject differences ^89^. Consequently, there will be greater statistical variance because of inter-subject variability in cell phenotype for a personalized cell therapy. Unlike clinical trials that use small and large molecules, the only source of statistical variability is the test subject’s clinical response to a drug. In contrast, there are two sources of statistical variability in autologous cell therapy – the treatment response and the test agent. A previous report identified genetic and non-genetic determinants as the source of these inter-subject differences in iPSC lines ^90^.

Allogeneic iPSC offers several advantages. First, the healthcare cost per patient is much less expensive than for an autologous cell therapy. Second, the inter-subject statistical variable for the cell phenotype is absent compared to autologous cell therapy since it is derived from a single tissue source. Neonatal sources like human foreskin fibroblasts, umbilical cord blood, umbilical cord tissue and placental tissue represent ideal target sources of somatic cell and stem cells to reprogram because they pose a much lower risk of somatic mutations. Moreover, cell heterogeneity among postnatal cells would be lower than adult cells in which the latter had more time to change from age, illness and environmental effects.

The main disadvantage of allogeneic cell therapy is that there is a HLA-mismatch between recipient and donor. Under these conditions, immunosuppressant agents are required to avoid host vs graft rejection. Yet, immunosuppressant drugs carry adverse clinical risks that include an increased risk of infection ^91,92^. To avoid the risk of immunosuppressant agents, there have been several efforts to use gene-editing techniques to knock-out MHC-1 and MHC-2 antigens to avoid T-cell-dependent adaptive immune responses ^93^. Yet, knock-out of the MHC-1 antigen evokes an innate cytotoxic immune response from NKC. Several strategies to avoid NKC-dependent immune responses include gene-editing knock-in of HLA-E, HLA-G, CD47 or membrane-bound and secreted β2m-HLA-G fusion protein ^92–97^.

However, universal allogeneic-iPSC therapies pose a potentially increased neoplastic risk than their original parental iPSC cell lines if target cells are reprogrammed with oncogenes. Host vs graft rejection represents a protected mechanism for immunological clearance of undesired foreign tissue. Yet, the neoplastic risk of an immune-tolerant universal iPSC therapy increases if undifferentiated cells remain in the cell product and could evade host immune clearance mechanisms. A possible solution would be to integrate into the genome an inducible gene switch that “turns off” cell therapy in the event a serious side effect emerges such as CAR-T-mediated cytokine storm ^98^. However, such inducible safety switches may be less effective for cell replacement therapy for preventing tumor formation since there is a lag time between tumor formation and diagnosis, and a delay in diagnosis could reduce the effectiveness of a genetically engineered switch.

### Engraftment Challenges of Induced Pluripotent Stem Cell-Dependent Cell Replacement Therapy

Induced pluripotent stem cell-derived cell replacement therapy is dependent on the ability of differentiated cells to engraft to previously diseased tissue environments. However, the therapeutic challenge for a successful cell replacement therapy is that the transplanted cells are subjected to the same inflammatory environment from chronic disease that caused the destruction of the original specialized cells ^99–103^. That state of local inflammation still persists, and transplanted cells would be subjected to the same inflammatory environment, which could cause the transplanted cells to fail to engraft or that the engraftment is short-lived. Thus, an important attribute for iPSC-derived cell replacement therapies to successfully engraft and maintain sufficient viability will be their capacity to resist local tissue inflammation.

## Conclusions

Induced pluripotent stem cells represent a promising new field where it may offer novel treatments for current unmet medical needs. However, the iPSC field also pose several technical challenges that are absent with traditional small and large molecules. Before iPSC-derived cell replacement therapies can be realized, several scientific, financial, manufacturing and regulatory challenges will ultimately need to be addressed. Those challenges include addressing genetic stability and cell heterogeneity, creating processes that improve cell safety, establishing manufacturing workflows that maintain cell reproducibility through the entire scale-up process, and implementing quality controls that characterize and predict cell potency. These milestones will be arduous to achieve. Yet, it is hopeful with persistence this field will finally deliver products that offer hope to patients who suffer from chronic disease.

## Figures and Tables

**Table 1:** Estimated Costs of Using Supply Chain Growth Factors to Manufacture Neuroprogenitor Cells for Different Levels of Scale-Up

		70 million NPC	700 million NPC	7 billion NPC
Growth Factor	Peptide Mass (μg)	Estimated Supply Chain Cost	Estimated Supply Chain Cost	Estimated Supply Chain Cost
Activin A	8000	$62,136.00	$621,360.00	$6,213,600.00
Wnt3A	8000	$66,052.00	$660,520.00	$6,605,200.00
Noggin	8000	$38,220.00	$382,200.00	$3,822,000.00
BDNF	8000	$45,247.00	$452,470.00	$4,524,700.00
**Total Cost**		**$211,655.00**	**$2,116,550.00**	**$21,165,500.00**

Table represents the estimated third-party supply chain cost of neural growth factors required to produce 70 million, 700 million and 7 billion neuroprogenitor cells (NPC). The minimum mass of each growth factor was produced from immortalized human somatic stem cells to differentiate 100 million iPSC into an estimated 70 million NPC based on expression of Nestin. iPSC differentiation into NPC was accomplished in three separate phases: (1) first neural rosette stage; (2) second neural rosette stage and (3) NPC induction stage. The estimated cost reflects the cost of growth factors if commercial third-party supply chains were instead used. The total estimated cost to produce 700 and 7 billion NPC is calculated by increasing the growth factor costs by a factor of 10 and 100 respectively.

## References

[1] Gerteis J ID, Deitz D, LeRoy L, Ricciardi R, Miller T, Basu J. Multiple Chronic Conditions Chartbook. Agency for Healthcare Research and Quality. 2014;April

[2] Khan AM, Green RS, Lytrivi ID, Sahulee R. Donor predictors of allograft utilization for pediatric heart transplantation. Transpl Int. Dec 2016;29(12):1269–1275. doi:10.1111/tri.12835 27542078

[3] Thiessen C, Kulkarni S, Reese PP, Gordon EJ. A Call for Research on Individuals Who Opt Out of Living Kidney Donation: Challenges and Opportunities. Transplantation. Dec 2016;100(12):2527–2532. doi:10.1097/tp.0000000000001408 27495760

[4] Thomson J, Itskovitz-Eldor J, Shapiro S, Embryonic stem cell lines derived from human blastocysts. Science. 1998;282:1827. 10.1126/science.282.5391.1145 9804556

[5] Baker D, Hirst AJ, Gokhale PJ, Detecting Genetic Mosaicism in Cultures of Human Pluripotent Stem Cells. Stem Cell Reports. Nov 8 2016;7(5):998–1012. doi:10.1016/j.stemcr.2016.10.003 27829140 PMC5106530

[6] Turinetto V, Orlando L, Giachino C. Induced Pluripotent Stem Cells: Advances in the Quest for Genetic Stability during Reprogramming Process. Int J Mol Sci. Sep 13 2017;18(9)doi:10.3390/ijms18091952 PMC5618601 28902128

[7] Van Voorhis BJ, Grinstead DM, Sparks AE, Gerard JL, Weir RF. Establishment of a successful donor embryo program: medical, ethical, and policy issues. Fertil Steril. Apr 1999;71(4):604–8. doi:10.1016/s0015-0282(98)00545-7 10202866

[8] Crook JM, Peura TT, Kravets L, The generation of six clinical-grade human embryonic stem cell lines. Cell Stem Cell. Nov 2007;1(5):490–4. doi:10.1016/j.stem.2007.10.004 18938745

[9] Patel SJ, Yamauchi T, Ito F. Induced Pluripotent Stem Cell-Derived T Cells for Cancer Immunotherapy. Surg Oncol Clin N Am. Jul 2019;28(3):489–504. doi:10.1016/j.soc.2019.02.005 31079802 PMC6516087

[10] Cichocki F, van der Stegen SJC, Miller JS. Engineered and banked iPSCs for advanced NK- and T-cell immunotherapies. Blood. Feb 23 2023;141(8):846–855. doi:10.1182/blood.2022016205 36327161 PMC10023718

[11] Maddineni S, Silberstein JL, Sunwoo JB. Emerging NK cell therapies for cancer and the promise of next generation engineering of iPSC-derived NK cells. J Immunother Cancer. May 2022;10(5)doi:10.1136/jitc-2022-004693 PMC9115029 35580928

[12] Netsrithong R, Wattanapanitch M. Advances in Adoptive Cell Therapy Using Induced Pluripotent Stem Cell-Derived T Cells. Front Immunol. 2021;12:759558. doi:10.3389/fimmu.2021.759558 34650571 PMC8505955

[13] Takahashi K, Tanabe K, Ohnuki M, Induction of Pluripotent Stem Cells from Adult Human Fibroblasts by Defined Factors. Cell. 2007;131(November 30):861–872. 18035408 10.1016/j.cell.2007.11.019

[14] Takahashi K, Yamanaka S. Induction of pluripotent stem cells from mouse embryonic and adult fibroblast cultures by defined factors. Cell. 2006;126:663–676. 16904174 10.1016/j.cell.2006.07.024

[15] Yu J, Vodyanik MA, Smuga-Otto K, Induced pluripotent stem cell lines derived from human somatic cells. Science. Dec 21 2007;318(5858):1917–20. doi:10.1126/science.1151526 18029452

[16] Nakagawa M, Koyanagi M, Tanabe K, Generation of induced pluripotent stem cells without Myc from mouse and human fibroblasts. Nat Biotechnol. Jan 2008;26(1):101–6. doi:10.1038/nbt1374 18059259

[17] Nakagawa M, Takizawa N, Narita M, Ichisaka T, Yamanaka S. Promotion of direct reprogramming by transformation-deficient Myc. Proc Natl Acad Sci U S A. Aug 10 2010;107(32):14152–7. doi:10.1073/pnas.1009374107 20660764 PMC2922531

[18] Ikegaki N, Minna J, Kennett RH. The human L-myc gene is expressed as two forms of protein in small cell lung carcinoma cell lines: detection by monoclonal antibodies specific to two myc homology box sequences. Embo j. Jun 1989;8(6):1793–9. doi:10.1002/j.1460-2075.1989.tb03573.x 2548855 PMC401025

[19] Bektas-Kayhan K, Unür M, Yaylim-Eraltan I, Role of L-MYC polymorphism in oral squamous cell carcinoma in Turkey. Anticancer Res. Jul 2009;29(7):2519–24. 19596922

[20] Yaylim-Eraltan I, Bozkurt N, Ergen A, L-myc gene polymorphism and risk of thyroid cancer. Exp Oncol. Jun 2008;30(2):117–20. 18566574

[21] Woltjen K, Michael IP, Mohseni P, piggyBac transposition reprograms fibroblasts to induced pluripotent stem cells. Nature. Apr 9 2009;458(7239):766–70. doi:10.1038/nature07863 19252478 PMC3758996

[22] Jia F, Wilson KD, Sun N, A nonviral minicircle vector for deriving human iPS cells. Nat Methods. Mar 2010;7(3):197–9. doi:10.1038/nmeth.1426 20139967 PMC2892897

[23] Anokye-Danso F, Trivedi CM, Juhr D, Highly efficient miRNA-mediated reprogramming of mouse and human somatic cells to pluripotency. Cell Stem Cell. Apr 8 2011;8(4):376–88. doi:10.1016/j.stem.2011.03.001 21474102 PMC3090650

[24] Fujie Y, Fusaki N, Katayama T, New type of Sendai virus vector provides transgene-free iPS cells derived from chimpanzee blood. PLoS One. 2014;9(12):e113052. doi:10.1371/journal.pone.0113052 25479600 PMC4257541

[25] Isono K, Jono H, Ohya Y, Generation of familial amyloidotic polyneuropathy-specific induced pluripotent stem cells. Stem Cell Res. Mar 2014;12(2):574–83. doi:10.1016/j.scr.2014.01.004 24531302

[26] Kawagoe S, Higuchi T, Otaka M, Morphological features of iPS cells generated from Fabry disease skin fibroblasts using Sendai virus vector (SeVdp). Mol Genet Metab. Aug 2013;109(4):386–9. doi:10.1016/j.ymgme.2013.06.003 23810832

[27] Yang W, Mills JA, Sullivan S, Liu Y, French DL, Gadue P. iPSC Reprogramming from Human Peripheral Blood Using Sendai Virus Mediated Gene Transfer. StemBook. Harvard Stem Cell Institute. Copyright: © 2012 Wenli Yang, Jason A. Mills, Spencer Sullivan, Ying Liu, Deborah L. French, and Paul Gadue.; 2008. 23785736

[28] Rosa A, Brivanlou AH. Synthetic mRNAs: powerful tools for reprogramming and differentiation of human cells. Cell Stem Cell. Nov 5 2010;7(5):549–50. doi:10.1016/j.stem.2010.10.002 21040893

[29] Warren L, Manos PD, Ahfeldt T, Highly efficient reprogramming to pluripotency and directed differentiation of human cells with synthetic modified mRNA. Cell Stem Cell. Nov 5 2010;7(5):618–30. doi:10.1016/j.stem.2010.08.012 20888316 PMC3656821

[30] Mandal PK, Rossi DJ. Reprogramming human fibroblasts to pluripotency using modified mRNA. Nat Protoc. Mar 2013;8(3):568–82. doi:10.1038/nprot.2013.019 23429718

[31] Yoshioka N, Gros E, Li HR, Efficient generation of human iPSCs by a synthetic self-replicative RNA. Cell Stem Cell. Aug 1 2013;13(2):246–54. doi:10.1016/j.stem.2013.06.001 23910086 PMC3845961

[32] Okita K, Matsumura Y, Sato Y, A more efficient method to generate integration-free human iPS cells. Nat Methods. May 2011;8(5):409–12. doi:10.1038/nmeth.1591 21460823

[33] Yu J, Hu K, Smuga-Otto K, Human induced pluripotent stem cells free of vector and transgene sequences. Science. May 8 2009;324(5928):797–801. doi:10.1126/science.1172482 19325077 PMC2758053

[34] Sullivan S, Stacey GN, Akazawa C, Quality control guidelines for clinical-grade human induced pluripotent stem cell lines. Regen Med. Oct 2018;13(7):859–866. doi:10.2217/rme-2018-0095 30205750

[35] Yin X, Li Y, Li J, Generation and periodontal differentiation of human gingival fibroblasts-derived integration-free induced pluripotent stem cells. Biochem Biophys Res Commun. May 6 2016;473(3):726–32. doi:10.1016/j.bbrc.2015.10.012 26456649

[36] Zhao T, Zhang ZN, Rong Z, Xu Y. Immunogenicity of induced pluripotent stem cells. Nature. May 13 2011;474(7350):212–5. doi:10.1038/nature10135 21572395

[37] Kamath A, Ternes S, McGowan S, English A, Mallampalli R, Moy AB. Efficient method to create integration-free, virus-free, Myc and Lin28-free human induced pluripotent stem cells from adherent cells. Future Sci OA. Aug 2017;3(3):Fso211. doi:10.4155/fsoa-2017-0028 PMC5583652 28884008

[38] Kamath A, Ternes S, McGowan S, Moy AB. Virus-free and oncogene-free induced pluripotent stem cell reprogramming in cord blood and peripheral blood in patients with lung disease. Regen Med. Dec 2018;13(8):889–915. doi:10.2217/rme-2018-0041 30488785

[39] Lee AS, Tang C, Cao F, Effects of cell number on teratoma formation by human embryonic stem cells. Cell Cycle. Aug 15 2009;8(16):2608–12. doi:10.4161/cc.8.16.9353 19597339 PMC2866168

[40] Pasi CE, Dereli-Öz A, Negrini S, Genomic instability in induced stem cells. Cell Death Differ. May 2011;18(5):745–53. doi:10.1038/cdd.2011.9 21311564 PMC3079512

[41] Kang X, Yu Q, Huang Y, Effects of Integrating and Non-Integrating Reprogramming Methods on Copy Number Variation and Genomic Stability of Human Induced Pluripotent Stem Cells. PLoS One. 2015;10(7):e0131128. doi:10.1371/journal.pone.0131128 26131765 PMC4488894

[42] Hawkins FJ, Suzuki S, Beermann ML, Derivation of Airway Basal Stem Cells from Human Pluripotent Stem Cells. Cell Stem Cell. Jan 7 2021;28(1):79–95.e8. doi:10.1016/j.stem.2020.09.017 33098807 PMC7796997

[43] Jacob A, Morley M, Hawkins F, Differentiation of Human Pluripotent Stem Cells into Functional Lung Alveolar Epithelial Cells. Cell Stem Cell. Oct 5 2017;21(4):472–488.e10. doi:10.1016/j.stem.2017.08.014 28965766 PMC5755620

[44] Antonov SA, Novosadova EV. Current State-of-the-Art and Unresolved Problems in Using Human Induced Pluripotent Stem Cell-Derived Dopamine Neurons for Parkinson’s Disease Drug Development. Int J Mol Sci. Mar 25 2021;22(7)doi:10.3390/ijms22073381 PMC8037675 33806103

[45] Bianchi F, Malboubi M, Li Y, Rapid and efficient differentiation of functional motor neurons from human iPSC for neural injury modelling. Stem Cell Res. Oct 2018;32:126–134. doi:10.1016/j.scr.2018.09.006 30278374

[46] Toba Y, Deguchi S, Mimura N, Comparison of commercially available media for hepatic differentiation and hepatocyte maintenance. PLoS One. 2020;15(2):e0229654. doi:10.1371/journal.pone.0229654 32106262 PMC7046223

[47] Li Y, Yang X, Plummer R, Human Pluripotent Stem Cell-Derived Hepatocyte-Like Cells and Organoids for Liver Disease and Therapy. Int J Mol Sci. Sep 28 2021;22(19)doi:10.3390/ijms221910471 PMC8508923 34638810

[48] Raju TS, Briggs JB, Borge SM, Jones AJ. Species-specific variation in glycosylation of IgG: evidence for the species-specific sialylation and branch-specific galactosylation and importance for engineering recombinant glycoprotein therapeutics. Glycobiology. May 2000;10(5):477–86. doi:10.1093/glycob/10.5.477 10764836

[49] Wang Y, Wu Z, Hu W, Hao P, Yang S. Impact of Expressing Cells on Glycosylation and Glycan of the SARS-CoV-2 Spike Glycoprotein. ACS Omega. Jun 22 2021;6(24):15988–15999. doi:10.1021/acsomega.1c01785 34179644 PMC8204757

[50] Goh JB, Ng SK. Impact of host cell line choice on glycan profile. Crit Rev Biotechnol. Sep 2018;38(6):851–867. doi:10.1080/07388551.2017.1416577 29262720

[51] Kaushik S, Mohanty D, Surolia A. Role of glycosylation in structure and stability of Erythrina corallodendron lectin (EcorL): a molecular dynamics study. Protein Sci. Mar 2011;20(3):465–81. doi:10.1002/pro.578 21432931 PMC3064827

[52] Kayser V, Chennamsetty N, Voynov V, Forrer K, Helk B, Trout BL. Glycosylation influences on the aggregation propensity of therapeutic monoclonal antibodies. Biotechnol J. Jan 2011;6(1):38–44. doi:10.1002/biot.201000091 20949542

[53] Li H, d’Anjou M. Pharmacological significance of glycosylation in therapeutic proteins. Curr Opin Biotechnol. Dec 2009;20(6):678–84. doi:10.1016/j.copbio.2009.10.009 19892545

[54] Öberg F, Sjöhamn J, Fischer G, Glycosylation increases the thermostability of human aquaporin 10 protein. J Biol Chem. Sep 9 2011;286(36):31915–23. doi:10.1074/jbc.M111.242677 21733844 PMC3173105

[55] Opanasopit P, Shirashi K, Nishikawa M, Yamashita F, Takakura Y, Hashida M. In vivo recognition of mannosylated proteins by hepatic mannose receptors and mannan-binding protein. Am J Physiol Gastrointest Liver Physiol. May 2001;280(5):G879–89. doi:10.1152/ajpgi.2001.280.5.G879 11292596

[56] Rajagopalan L, Organ-Darling LE, Liu H, Glycosylation regulates prestin cellular activity. J Assoc Res Otolaryngol. Mar 2010;11(1):39–51. doi:10.1007/s10162-009-0196-5 19898896 PMC2820205

[57] Straumann N, Wind A, Leuenberger T, Wallimann T. Effects of N-linked glycosylation on the creatine transporter. Biochem J Jan 15 2006;393(Pt 2):459–69. doi:10.1042/bj20050857 16167890 PMC1360696

[58] Su D, Zhao H, Xia H. Glycosylation-modified erythropoietin with improved half-life and biological activity. Int J Hematol. Mar 2010;91(2):238–44. doi:10.1007/s12185-010-0496-x 20131103

[59] Arai S, Shibazaki C, Adachi M, The non-glycosylated N-terminal domain of human thrombopoietin is a molten globule under native conditions. Febs j. May 2019;286(9):1717–1733. doi:10.1111/febs.14765 30675759

[60] Costamagna D, Mommaerts H, Sampaolesi M, Tylzanowski P. Noggin inactivation affects the number and differentiation potential of muscle progenitor cells in vivo. Sci Rep. Aug 30 2016;6:31949. doi:10.1038/srep31949 27573479 PMC5004166

[61] Komekado H, Yamamoto H, Chiba T, Kikuchi A. Glycosylation and palmitoylation of Wnt-3a are coupled to produce an active form of Wnt-3a. Genes Cells. Apr 2007;12(4):521–34. doi:10.1111/j.1365-2443.2007.01068.x 17397399

[62] Dumont J, Euwart D, Mei B, Estes S, Kshirsagar R. Human cell lines for biopharmaceutical manufacturing: history, status, and future perspectives. Crit Rev Biotechnol. Dec 2016;36(6):1110–1122. doi:10.3109/07388551.2015.1084266 26383226 PMC5152558

[63] Wong A The ethics of HEK 293. Natl Cathol Bioeth Q. Autumn 2006;6(3):473–95. doi:10.5840/ncbq20066331 17091554

[64] Lin YC, Boone M, Meuris L, Genome dynamics of the human embryonic kidney 293 lineage in response to cell biology manipulations. Nat Commun. Sep 3 2014;5:4767. doi:10.1038/ncomms5767 25182477 PMC4166678

[65] Graham FL, Smiley J, Russell WC, Nairn R. Characteristics of a human cell line transformed by DNA from human adenovirus type 5. J Gen Virol. Jul 1977;36(1):59–74. doi:10.1099/0022-1317-36-1-59 886304

[66] Louis N, Evelegh C, Graham FL. Cloning and sequencing of the cellular-viral junctions from the human adenovirus type 5 transformed 293 cell line. Virology. Jul 7 1997;233(2):423–9. doi:10.1006/viro.1997.8597 9217065

[67] Croset A, Delafosse L, Gaudry JP, Differences in the glycosylation of recombinant proteins expressed in HEK and CHO cells. J Biotechnol. Oct 31 2012;161(3):336–48. doi:10.1016/j.jbiotec.2012.06.038 22814405

[68] Böhm E, Seyfried BK, Dockal M, Differences in N-glycosylation of recombinant human coagulation factor VII derived from BHK, CHO, and HEK293 cells. BMC Biotechnol. Sep 18 2015;15:87. doi:10.1186/s12896-015-0205-1 26382581 PMC4574471

[69] Fan Y, Winanto Ng SY. Replacing what’s lost: a new era of stem cell therapy for Parkinson’s disease. Transl Neurodegener. 2020;9:2. doi:10.1186/s40035-019-0180-x 31911835 PMC6945567

[70] Kim J, Jeon J, Song B, Spotting-based differentiation of functional dopaminergic progenitors from human pluripotent stem cells. Nat Protoc. Mar 2022;17(3):890–909. doi:10.1038/s41596-021-00673-4 35140411 PMC9511827

[71] 21 CFR Part 1271 (Human Cells, Tissues, and Cellular and Tissue-Based Products). https://wwwecfrgov/current/title-21/chapter-I/subchapter-L/part-1271.

[72] 21 Part 600 (Biological Products: General) https://wwwecfrgov/current/title-21/chapter-I/subchapter-F/part-600.

[73] 21 CFR Part 610 (General Biological Products Standards). https://wwwecfrgov/current/title-21/chapter-I/subchapter-F/part-610.

[74] 21 CFR Part 211 (Current Good Manufacturing Practice). https://wwwecfrgov/current/title-21/chapter-I/subchapter-C/part-211.

[75] Guideline on quality, non-clinical and clinical aspects of medicinal products containing genetically modified cells. https://wwwemaeuropaeu/en/documents/scientific-guideline/guideline-quality-non-clinical-clinical-aspects-medicinal-products-containing-genetically-modified_en-0pdf. 10.1111/bcp.16047 38565322

[76] Baust JG, Snyder KK, Van Buskirk R, Baust JM. Integrating Molecular Control to Improve Cryopreservation Outcome. Biopreserv Biobank. Apr 2017;15(2):134–141. doi:10.1089/bio.2016.0119 28332850

[77] Baust JM, Corwin W, Snyder KK, Van Buskirk R, Baust JG. Cryopreservation: Evolution of Molecular Based Strategies. Adv Exp Med Biol. 2016;951:13–29. doi:10.1007/978-3-319-45457-3_2 27837551

[78] Baust JM, Corwin WL, VanBuskirk R, Baust JG. Biobanking: The Future of Cell Preservation Strategies. Adv Exp Med Biol. 2015;864:37–53. doi:10.1007/978-3-319-20579-3_4 26420612

[79] Baust JM, Vogel MJ, Van Buskirk R, Baust JG. A molecular basis of cryopreservation failure and its modulation to improve cell survival. Cell Transplant. 2001;10(7):561–71. 11714190

[80] Myles L, Church TD. An industry survey of implementation strategies for clinical supply chain management of cell and gene therapies. Cytotherapy. Mar 2022;24(3):344–355. doi:10.1016/j.jcyt.2021.09.012 34750073

[81] Weltner J, Balboa D, Katayama S, Human pluripotent reprogramming with CRISPR activators. Nat Commun. Jul 6 2018;9(1):2643. doi:10.1038/s41467-018-05067-x 29980666 PMC6035213

[82] Dominguez AA, Lim WA, Qi LS. Beyond editing: repurposing CRISPR-Cas9 for precision genome regulation and interrogation. Nat Rev Mol Cell Biol. Jan 2016;17(1):5–15. doi:10.1038/nrm.2015.2 26670017 PMC4922510

[83] Hockemeyer D, Jaenisch R. Induced Pluripotent Stem Cells Meet Genome Editing. Cell Stem Cell. May 5 2016;18(5):573–86. doi:10.1016/j.stem.2016.04.013 27152442 PMC4871596

[84] Ding Q, Regan SN, Xia Y, Oostrom LA, Cowan CA, Musunuru K. Enhanced efficiency of human pluripotent stem cell genome editing through replacing TALENs with CRISPRs. Cell Stem Cell. Apr 4 2013;12(4):393–4. doi:10.1016/j.stem.2013.03.006 23561441 PMC3925309

[85] Bhardwaj A, Nain V. TALENs-an indispensable tool in the era of CRISPR: a mini review. J Genet Eng Biotechnol. Aug 21 2021;19(1):125. doi:10.1186/s43141-021-00225-z 34420096 PMC8380213

[86] Mussolino C, Alzubi J, Fine EJ, TALENs facilitate targeted genome editing in human cells with high specificity and low cytotoxicity. Nucleic Acids Res. Jun 2014;42(10):6762–73. doi:10.1093/nar/gku305 24792154 PMC4041469

[87] Dhatchinamoorthy K, Colbert JD, Rock KL. Cancer Immune Evasion Through Loss of MHC Class I Antigen Presentation. Front Immunol. 2021;12:636568. doi:10.3389/fimmu.2021.636568 33767702 PMC7986854

[88] Volpato V, Webber C. Addressing variability in iPSC-derived models of human disease: guidelines to promote reproducibility. Dis Model Mech. Jan 17 2020;13(1)doi:10.1242/dmm.042317 PMC6994963 31953356

[89] Kilpinen H, Goncalves A, Leha A, Common genetic variation drives molecular heterogeneity in human iPSCs. Nature. Jun 15 2017;546(7658):370–375. doi:10.1038/nature22403 28489815 PMC5524171

[90] Carcamo-Orive I, Hoffman GE, Cundiff P, Analysis of Transcriptional Variability in a Large Human iPSC Library Reveals Genetic and Non-genetic Determinants of Heterogeneity. Cell Stem Cell. Apr 6 2017;20(4):518–532.e9. doi:10.1016/j.stem.2016.11.005 28017796 PMC5384872

[91] Meneghini M, Bestard O, Grinyo JM. Immunosuppressive drugs modes of action. Best Pract Res Clin Gastroenterol. Oct-Dec 2021;54–55:101757. doi:10.1016/j.bpg.2021.101757 34874841

[92] Deuse T, Hu X, Gravina A, Hypoimmunogenic derivatives of induced pluripotent stem cells evade immune rejection in fully immunocompetent allogeneic recipients. Nat Biotechnol. Mar 2019;37(3):252–258. doi:10.1038/s41587-019-0016-3 30778232 PMC6419516

[93] Mattapally S, Pawlik KM, Fast VG, Human Leukocyte Antigen Class I and II Knockout Human Induced Pluripotent Stem Cell-Derived Cells: Universal Donor for Cell Therapy. J Am Heart Assoc. Dec 4 2018;7(23):e010239. doi:10.1161/jaha.118.010239 30488760 PMC6405542

[94] Han X, Huang H, Gao P, E-protein regulatory network links TCR signaling to effector Treg cell differentiation. Proc Natl Acad Sci U S A. Mar 5 2019;116(10):4471–4480. doi:10.1073/pnas.1800494116 30770454 PMC6410771

[95] Gornalusse GG, Hirata RK, Funk SE, HLA-E-expressing pluripotent stem cells escape allogeneic responses and lysis by NK cells. Nat Biotechnol. Aug 2017;35(8):765–772. doi:10.1038/nbt.3860 28504668 PMC5548598

[96] Ye Q, Sung TC, Yang JM, Ling QD, He Y, Higuchi A. Generation of universal and hypoimmunogenic human pluripotent stem cells. Cell Prolif. Dec 2020;53(12):e12946. doi:10.1111/cpr.12946 33174655 PMC7705897

[97] Shi L, Li W, Liu Y, Generation of hypoimmunogenic human pluripotent stem cells via expression of membrane-bound and secreted β2m-HLA-G fusion proteins. Stem Cells. Nov 2020;38(11):1423–1437. doi:10.1002/stem.3269 32930470

[98] Chakravarti D, Caraballo LD, Weinberg BH, Wong WW. Inducible Gene Switches with Memory in Human T Cells for Cellular Immunotherapy. ACS Synth Biol. Aug 16 2019;8(8):1744–1754. doi:10.1021/acssynbio.8b00512 31268301 PMC6703182

[99] Stephenson J, Nutma E, van der Valk P, Amor S. Inflammation in CNS neurodegenerative diseases. Immunology. Jun 2018;154(2):204–219. doi:10.1111/imm.12922 PMC5980185 29513402

[100] Arévalo NB, Lamaizon CM, Cavieres VA, Neuronopathic Gaucher disease: Beyond lysosomal dysfunction. Front Mol Neurosci. 2022;15:934820. doi:10.3389/fnmol.2022.934820 35992201 PMC9381931

[101] Francelle L, Mazzulli JR. Neuroinflammation in Gaucher disease, neuronal ceroid lipofuscinosis, and commonalities with Parkinson’s disease. Brain Res. Apr 1 2022;1780:147798. doi:10.1016/j.brainres.2022.147798 35063468 PMC9126024

[102] Sheehy DF, Quinnell SP, Vegas AJ. Targeting Type 1 Diabetes: Selective Approaches for New Therapies. Biochemistry. Jan 29 2019;58(4):214–233. doi:10.1021/acs.biochem.8b01118 30608114 PMC6774349

[103] Monti P, Vignali D, Piemonti L. Monitoring Inflammation, Humoral and Cell-mediated Immunity in Pancreas and Islet Transplants. Curr Diabetes Rev. 2015;11(3):135–43. doi:10.2174/1573399811666150317125820 25777058 PMC5398085

